# Sustaining progress towards universal health coverage amidst a full-scale war: learning from Ukraine

**DOI:** 10.1093/heapol/czae041

**Published:** 2024-06-08

**Authors:** Jarno Habicht, Mark Hellowell, Joe Kutzin

**Affiliations:** World Health Organization Office for Ukraine, Kyiv, Ukraine; School of Social and Political Science, University of Edinburgh, Edinburgh, United Kingdom; Independent Consultant

**Keywords:** Health financing, conflict, resilience, Ukraine, purchasing

## Abstract

In the aftermath of Russia’s military response to the 2014 Revolution of Dignity, the government of Ukraine implemented a package of health financing reforms underpinned by universal health coverage (UHC) principles. By the time of Russia’s full-scale invasion of Ukraine in February 2022, the new systems and institutions envisaged in the reforms were largely established. In this *Commentary* article, we explain how these attributes strengthened the Ukrainian health system’s response to the impacts of the war. Ukraine’s experience highlights the role that health financing arrangements, designed in accordance with UHC principles, can play in strengthening health system resilience.

Key messages

- In the aftermath of Russia’s military response to the 2014 Revolution of Dignity, the government of Ukraine implemented a package of health financing reforms aimed at universal health coverage (UHC).
- By the time of Russia’s full-scale, nationwide, invasion of Ukraine on 24 February 2022, these reforms had established: (i) a guaranteed benefits package for the whole population, funded by general government revenues; (ii) a new ‘single purchaser agency, the National Health Service of Ukraine; (iii) explicit budgetary prioritization of primary health care; (iv) a shift from paying health facilities for ‘inputs’ to paying them for patient coverage, or patient care and (v) an e-health information system, supporting the above.
- We argue that these systems and institutions strengthened Ukraine’s ability to maintain the core health system functions in the most conflict-affected regions, and lead effective responses to the wider economic, demographic and epidemiological impacts of the war.
- Ukraine’s experience highlights the role that health financing arrangements designed in accordance with UHC principles can play in strengthening health system resilience, even in the face of war.

In the aftermath of Russia’s military response to the 2014–15 Revolution of Dignity, the government of Ukraine implemented a package of health system reforms aimed at universal health coverage (UHC) (Dale *et al*., 2019; Dzhygyr *et al*., 2023). By the time of Russia’s full-scale, nationwide, invasion of Ukraine, in February 2022, the new health financing systems and institutions were in place.

In this *Commentary*, we explore how these strengthened Ukraine’s ability to maintain core health system functions across the country, including in the most conflict-affected areas, and mount effective responses to the wider economic, demographic and epidemiological shocks of the war. On this basis, we contend that, in its future strategies for reform, recovery and reconstruction, the government of Ukraine should hold fast to core UHC principles of equity and solidarity—with continued entitlement to publicly financed services based on residency, centralized pooling, strong purchasing arrangements and continued action to minimize user charges.

When, in response to the Revolution of Dignity, Russia invaded Crimea and fomented armed violence in the Donbas, leading to a protracted conflict, a deep recession and the displacement of 1.8 million people [United Nations Office for the Coordination of Humanitarian Affairs (OCHA), 2016] Ukraine’s health system was unprepared. Many features of the old Soviet ‘Semashko’ system remained in place (Lekhan *et al*., 2015). Funding was decentralized and services fragmented—with multiple payers and parallel structures of delivery (Davis, 2010). Funding for health facilities was input-based—designed to sustain infrastructure and not to meet the health care needs of the population. Accountability for performance was diffuse and unclear; and, in any case, no information system capable of monitoring performance existed.

In the conflict-affected areas of Donetsk and Luhansk, the central and regional authorities were unable to provide funding to sustain core functions, and many people lost access to basic services (Lekhan *et al*., 2015). Nominally, the whole population of Ukraine had the right to access health services in public facilities. But with increasingly tight government budget constraints, and no defined package of entitlements, this right could not be enforced in practice, and it often fell to providers to define the terms of access. In this context, the health needs of many displaced people went unmet—while others in need of care faced rising out-of-pocket, and often catastrophic, costs (Goroshko *et al*., 2018).

Such experiences only strengthened the impetus for reform, however. In 2016, the Cabinet of Ministers approved a new *Concept for Health Financing Reform* (Concept for Health Financing System Reform, 2024). The resulting legislation introduced a defined benefits package—including the Program of Medical Guarantees (PMG) and an Affordable Medicines Program (AMP). A proposal to fund these entitlements via contributory social health insurance was rejected, with the government opting instead for general government revenues—retaining the constitutional principle that all residents should be covered regardless of contribution (Concept for Health Financing System Reform, 2024). Moves were made to consolidate government funding and risk pools, with the majority of public funds to be managed by a ‘single payer’ agency, the National Health Service of Ukraine (NHSU), and directed to facilities according to capitation (in primary health care) and activity-based payments (in specialist and inpatient care), supported by a unified national e-health information system (Bredenkamp *et al*., 2018)^.^

These changes were accompanied by an increase in funding for primary care, strengthening the role and status of this important, but previously marginalized, component of the system (Bredenkamp *et al*., 2018). All Ukrainian residents were, for the first time, able to enrol with a family doctor of their choice, receiving from them a defined package of primary health care services, free-at-the-point-of-use. By the time of Russia’s full-scale invasion, on 24 February 2022, some 32.5 million Ukrainians (82% of the population) had enrolled (Goroshko *et al*., 2023). More broadly, despite the invasion and the further disruptions caused by the COVID-19 pandemic, Ukraine had made considerable progress in health financing reforms, creating the conditions for improved service coverage and financial protection (Kasekamp *et al*., 2023), especially in primary care (USAID Health Care Reform Support Project, 2021)^.^

For >2 years, Ukraine’s health system has operated in the midst of Europe’s largest conflict since the Second World War [Office of the High Commissioner for Human Rights (OHCHR, 2024]. As of March 2024, there have been 1682 confirmed attacks on health care in Ukraine, resulting in 128 deaths and 288 injuries (World Health Organization, 2024b). Of the 9925 health facilities in the country, some 1242 have been partially or fully damaged (World Bank, 2023a). The scale of population displacement is unprecedented in post-war Europe (World Bank, 2023a), while needs have increased in a range of areas, including emergency medical services, trauma and burns, rehabilitation and mental health conditions (World Bank, 2023a).

From the first months of the war, the new health financing institutions played a key role in the health system’s response: enhancing its capacities to prepare for and respond to conflict-related disruptions, maintain core functions and reorganize as conditions required—capacities that Kruk et al. influentially defined as the core attributes of resilience (Kruk *et al*., 2015).

By relying on general government revenues, rather than specific contributions, an insured/uninsured division in the population was avoided, and PMG and AMP legal entitlements for all residents maintained, mitigating the effects of the economic shock on access to services and medicines, including for displaced and vulnerable people. By the end of 2023, Ukraine’s economy was 26% smaller than in 2021 (World Bank, 2023b), resulting in a rise in the poverty rate from 5% in 2021 to 24% in 2022, and in unemployment—and, overall, major reductions to households’ ability to pay for care (World Bank, 2023a). Although financial protection has long been weaker in Ukraine than elsewhere in Europe, especially for poorer people (WHO Regional Office for Europe, 2023), a survey undertaken in 2023 found that 95% of people who sought care from family doctors were able to access it; and 93% of people who sought outpatient medicines received them (World Health Organization, 2024a). In 2023, an estimated 6.2 million Ukrainians were recorded as refugees, the majority in other European countries (Operational Data Portal, 2024) with 3.7 million internally displaced (International Organization for Migration (IOM), 2024). Such persons are more likely to report barriers to access—with challenges involving time, unavailability of services and lack of necessary documents—than the rest of the population. But guaranteed, non-contributory, entitlements for all Ukrainians ensures that those internally displaced are eligible to receive services in their host locations (WHO Regional Office for Europe, 2022).

In addition, centralization of pooling enabled continued availability of health services in territories facing diminishing locally generated revenues—including the regions closest to the front-line, and those liberated from Russian occupation (WHO, 2023). In these regions, access to care across most service domains has been sustained at a high level (Assaf *et al.*, 2024). Strengthening of purchasing supported the health system’s ability to adapt to changing circumstances. In early 2022, the NHSU shifted from capitation (for primary care facilities) and activity-based payments (for hospitals) to global budgets, allowing rapid disbursement of funds to pay for health workers’ salaries and other essential inputs in the war-affected regions—a shift that was then reversed, later in the year, in the regions in which conditions had stabilized. Such rapid adaptation would have been far more difficult under the old, rigid, input-based, line-item arrangements (Goroshko and Kasyanchuk, 2022). The more explicit definition of coverage under the PMG strengthened the response to new needs arising from the war—including in the form of new packages for mental health at the primary care level, and for complex inpatient rehabilitation services (United Nations OCHA, 2024).

Thus, the post-2016 reforms supported the health system’s role in both routine service delivery nationwide, and in the emergency response in the regions and for the populations most affected by war—with the humanitarian sector playing a critical but complementary role (United Nations OCHA, 2024).

Three years into the war, the challenges faced by the health system of Ukraine remain profound. Fiscal constraints are tightening. Emergency arrangements—to meet, for example, the health needs of displaced people—must now be placed on a sustainable footing. Service disruptions continue where hostilities are ongoing. Nationwide, long-standing inefficiencies in service delivery (including an excessive hospital infrastructure) and institutional weaknesses (including corruption and a diminishing, but still active, informal economy) must be addressed—especially in the context of ongoing EU accession talks (World Health Organization, World Bank, USAID, European Union, 2023).

In responding to these challenges, and in its broader approach to recovery and reconstruction, (World Health Organization, 2022) Ukraine should hold fast to core UHC principles of equity and solidarity—with continued entitlement to publicly financed health services based on residency, central pooling, and strong purchasing, alongside minimization of user charges. These attributes have so far enabled the health system to mitigate, if not fully address, the economic, demographic and epidemiological disruptions generated by the war - Europe’s largest since 1945. This experience holds important lessons for Ukraine, as the reforms continue to be implemented, and are even extended, despite of the war; and also for other countries, as they seek to enhance the resilience of their health systems at a time of increased geopolitical insecurity.

## Reflexivity statement

One of the authors (J.H.) is a Representative and Head of the WHO Country Office in Ukraine and is based in Kyiv. He has long-standing experience of working closely with the government of Ukraine and other stakeholders on addressing health system and health financing challenges, including both before and after the 2022 invasion. Two of the authors (M.H. and J.K.) are specialists in health financing and are based outside of the country—albeit with direct, extensive, experience of working in Ukraine.
